# Development of an Interoperable and Easily Transferable Clinical Decision Support System Deployment Platform: System Design and Development Study

**DOI:** 10.2196/37928

**Published:** 2022-07-27

**Authors:** Junsang Yoo, Jeonghoon Lee, Ji Young Min, Sae Won Choi, Joon-myoung Kwon, Insook Cho, Chiyeon Lim, Mi Young Choi, Won Chul Cha

**Affiliations:** 1 Department of Digital Health Samsung Advanced Institute for Health Science & Technology Sungkyunkwan University Seoul Republic of Korea; 2 HD Junction, Inc Seoul Republic of Korea; 3 LINEWALKS Inc Seoul Republic of Korea; 4 Office of Hospital Information Seoul National University Hospital Seoul Republic of Korea; 5 Medical Research Team Medical AI Inc Seoul Republic of Korea; 6 Nursing Department School of Medicine Inha University Incheon Republic of Korea; 7 Department of Biostatistics Dongguk University School of Medicine Goyang Republic of Korea; 8 Data Service Center en-core Co, Ltd Seoul Republic of Korea; 9 Department of Emergency Medicine Samsung Medical Center Sungkyunkwan University School of Medicine Seoul Republic of Korea; 10 Digital Innovation Center Samsung Medical Center Sungkyunkwan University School of Medicine Seoul Republic of Korea

**Keywords:** clinical decision support system, decision making, decision aid, decision support, common data model, model, development, electronic health record, medical record, EHR, EMR, Fast Healthcare Interoperability Resource, interoperability, machine learning, clinical decision, health technology, algorithm, intelligent algorithm network, modeling

## Abstract

**Background:**

A clinical decision support system (CDSS) is recognized as a technology that enhances clinical efficacy and safety. However, its full potential has not been realized, mainly due to clinical data standards and noninteroperable platforms.

**Objective:**

In this paper, we introduce the common data model–based intelligent algorithm network environment (CANE) platform that supports the implementation and deployment of a CDSS.

**Methods:**

CDSS reasoning engines, usually represented as R or Python objects, are deployed into the CANE platform and converted into C# objects. When a clinician requests CANE-based decision support in the electronic health record (EHR) system, patients’ information is transformed into Health Level 7 Fast Healthcare Interoperability Resources (FHIR) format and transmitted to the CANE server inside the hospital firewall. Upon receiving the necessary data, the CANE system’s modules perform the following tasks: (1) the preprocessing module converts the FHIRs into the input data required by the specific reasoning engine, (2) the reasoning engine module operates the target algorithms, (3) the integration module communicates with the other institutions’ CANE systems to request and transmit a summary report to aid in decision support, and (4) creates a user interface by integrating the summary report and the results calculated by the reasoning engine.

**Results:**

We developed a CANE system such that any algorithm implemented in the system can be directly called through the RESTful application programming interface when it is integrated with an EHR system. Eight algorithms were developed and deployed in the CANE system. Using a knowledge-based algorithm, physicians can screen patients who are prone to sepsis and obtain treatment guides for patients with sepsis with the CANE system. Further, using a nonknowledge-based algorithm, the CANE system supports emergency physicians’ clinical decisions about optimum resource allocation by predicting a patient’s acuity and prognosis during triage.

**Conclusions:**

We successfully developed a common data model–based platform that adheres to medical informatics standards and could aid artificial intelligence model deployment using R or Python.

## Introduction

The clinical decision support system (CDSS) is expected to play an essential role in modern medicine. The expansion of scalable data and advances in data science have led to considerable data-driven CDSS research, which has offered good opportunities to accurately reflect the clinical context with higher complexity than possible with rule-based expert systems [1]. The establishment of a research process for the development, validation, and reporting of machine-learning algorithms has made significant contributions to improving quality and reproducibility in this area [2,3].

Even the best algorithm cannot be expected to achieve its potential benefit before it is utilized in a clinical setting [4]. The transition of an algorithm from research to implementation is hindered by several factors, including social, political, economic, clinical, and technical issues [5-8]. Among these, the interoperability problem, which originates from the heterogeneity of electronic health record (EHR) systems with varying data types and structures, has been identified as an important factor that hinders CDSS implementation in a real clinical setting [9,10]. Moreover, considering that a current data-driven CDSS utilizes more variables than traditional statistical models and requires data preprocessing, it is unrealistic to expect CDSS developers to modify their model to fit each hospital’s EHR system.

Dozens of standards have been introduced to overcome this interoperability issue, including the International Statistical Classification of Diseases and Related Health Problems, 10th revision; the Logical Observation Identifiers Names and Codes taxonomy; the RxNorm drug vocabulary; and the SNOMED (Systematized Nomenclature of Medicine–Clinical Terms) clinical terminology database for semantic technology integration [11]. Additionally, Health Level 7 (HL7) V2 and V3 negotiated frameworks, clinical document architectures, and HL7 Fast Healthcare Interoperability Resources (FHIR) for data exchange [12,13] have been used. The Observational Medical Outcomes Partnership (OMOP) common data model (CDM), the Sentinel CDM, and the National Patient-Centered Clinical Outcomes Research Network (PCORnet) CDM for standardized data structures and types [14,15] are other major standards developments. However, because hospitals in South Korea utilize heterogeneous home-grown EHR systems, medical informaticians face consistent difficulties in adopting international medical data standards. More recently, Clinical Quality Language (CQL) and CDS Hooks were introduced [16,17]. CQL is a language that is used in various clinical situations, including clinical decision-making, cohort definition, and clinical quality measurements. CQL can be easily integrated into HL7 FHIR via sharing functions, which helps domain experts by enhancing human readability.

The OMOP-CDM and HL7 FHIR standards are good starting points for developing a platform that can deploy an interoperable CDSS to multiple organizations [18,19]. Moreover, the OMOP-CDM has acquired the status of a de facto standard in South Korea. Over two-thirds of tertiary academic hospitals have adopted the OMOP-CDM with national research and development support [20]. Additionally, HL7 FHIR is known as a prospering standard in the medical informatics field. This standard provides a simplified data model using the FHIR 80% rule. That is, the operative guideline informally states that each resource should contain only those data elements agreed upon by 80% or more of the participants in the development effort [13,21]. Because HL7 FHIR employs a web protocol, the standard is widely used to exchange information in a variety of medical settings, including those of CDSS deployments [10,18,19].

The objective of this study was to introduce the CDM-based intelligent algorithm network environment (CANE) platform to support the implementation and deployment of a CDSS.

## Methods

### CANE Research Consortium

The CANE Research Consortium was established in May 2019 to develop a CDSS deployment platform that could extend CDSS data referencing capabilities across medical institutions. The Consortium comprised six research groups representing seven major hospitals in Seoul, Gyeonggi, and Incheon, South Korea.

### CANE Architecture

**Figure 1:**
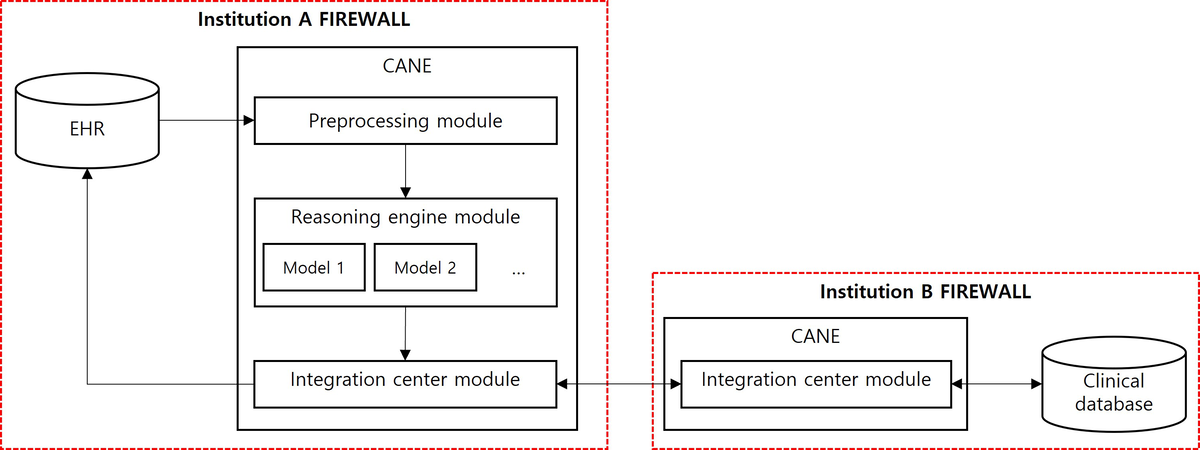
Abstract architecture of the common data model–based intelligent algorithm network environment (CANE) platform. EHR: electronic health record.

The CANE platform is built on the Linux (CentOS 7.7 (1906)) operating system with 3.7-GHz octa core CPUs, 64-GB RAM, and a 2-TB hard disk drive. Microsoft.Net 5.0, MariaDB 10.4.12 (x86_64), Python 3.6.8, and Apache 2.4.6 software systems are applied. The platform consists of a preprocessing module, a reasoning engine, and an integration center module. The roles of each module were described in the CDSS operation process session (Figure 1).

### Model Development, Deployment, and Operation Processes

#### Phase 1: Development and Evaluation of the CDSS Reasoning Engine

Algorithms distributed in CANE are classified into knowledge- and nonknowledge-based CDSSs. A knowledge-based CDSS refers to a traditional expert system that provides informational representations of medical guidelines. A nonknowledge-based CDSS uses machine learning, a technology that recognizes patterns and makes predictions from clinical data.

**Figure 2:**
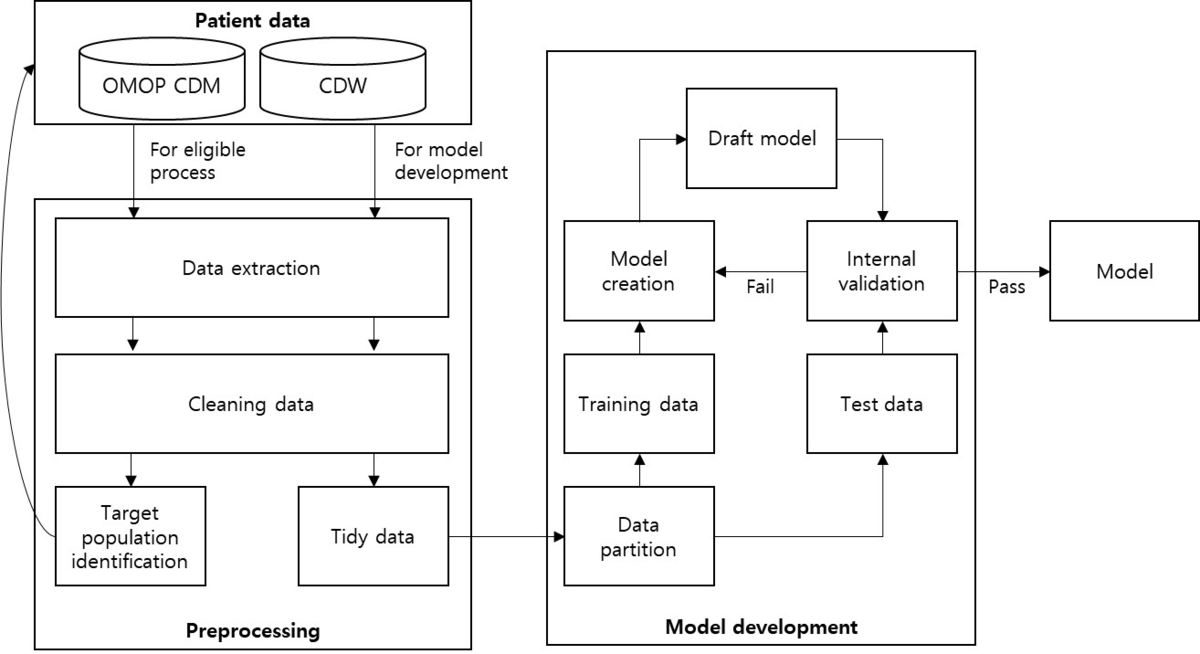
Nonknowledge-based clinical decision support system model development process. CDM: common data model; CDW: clinical data warehouse; OMOP: Observational Medical Outcome Partnership.

Figure 2 describes the development process of the nonknowledge-based CDSS. In principle, data for algorithm development should be extracted from the OMOP-CDM database. However, learning from a local clinical data warehouse is also allowed because it may be necessary to learn from data that cannot be converted into OMOP-CDM format. There are various ways to develop a machine-learning algorithm. In this study, we followed the patient-level prediction framework: (1) target population identification, (2) predictor extraction, (3) splitting tidy data into training and test sets, (4) draft model development using a training data set, (5) iterative process of evaluating the draft model, and (6) final model confirmation.

#### Phase 2: CDSS Deployment on the CANE Platform

**Figure 3:**
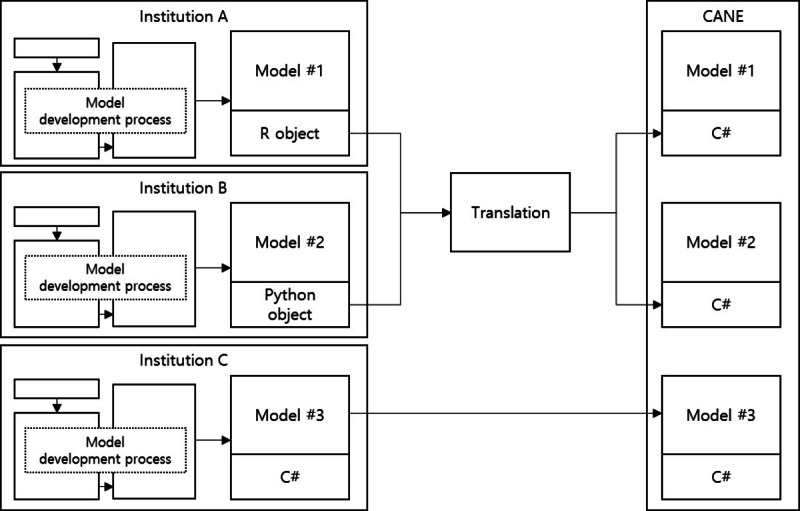
Model deployment process.

The developed algorithms usually take the form of R or Python objects, which are widely used by researchers in the field of medical informatics. Converting these objects into C# language is a prerequisite for mounting the reasoning engine module of the CANE platform (Figure 3). This model conversion process is performed using the Hl.Fhir.R4 (2.0.0), Newtonsoft.Json (12.0.3), and R.NET (1.9.0) packages. Moreover, the model formed of C# objects is simply deployed without conversion. By using C#, which is the most representative language of the .NET framework, programs can be executed on any operating system following the common language specification.

#### Phase 3: CDSS Operation Process

**Figure 4:**
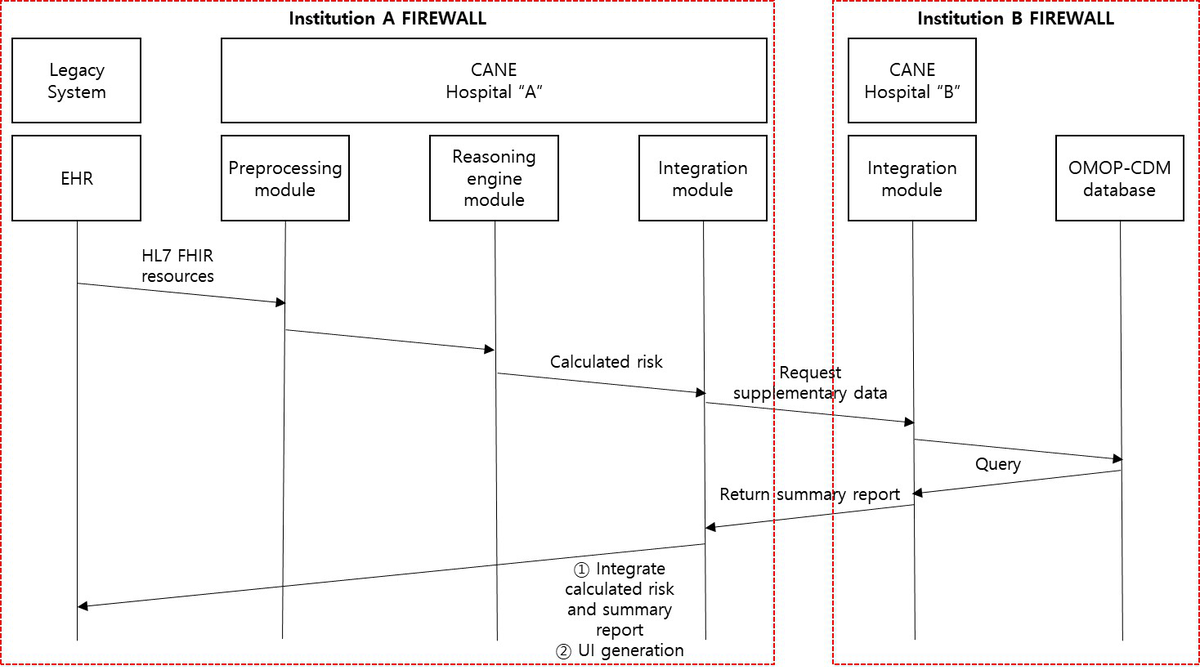
Clinical decision support system operation process with CANE. EHR: electronic health record; FHIR: Fast Healthcare Interoperability Resource; HL7: Health Level 7; OMOP-CDM: Observational Medical Outcome Partnership-common data model; UI: user interface.

A user requests decision support from the CANE platform by clicking the “CANE” button on their EHR user interface (UI). Subsequently, the EHR data required for the target algorithm are converted into JavaScript Object Notation (JSON) format using HL7 FHIRs. They are then transmitted to the CANE server. Next, the CANE server parses and converts the received FHIRs to fit the target CDS algorithm. The preprocessed input data call the deployed model, and the model returns the calculated score (eg, sepsis risk score) to the integration module. All information transmissions occur between the CANE and EHR, where these systems were implemented, without integration modules. The integration module requests additional information from other institutions’ CANE systems, which are interconnected via their own OMOP-CDM. To dispel potential privacy concerns, the data used for interinstitution transmission are delivered as a population-level summary rather than raw data that can be used to identify individuals. This summary data can assist the physician in possibly promoting patient behavioral changes in favorable ways. Finally, the integration module generates a UI based on the calculated score from the model and supplementary data from other institutions’ CANE systems, which are then presented to the EHR. These operating processes are described in Figure 4.

### Ethics Approval

The sepsis case study was approved by the Samsung Medical Center Institutional Review Board (2019-07-034) and the emergency department (ED) case study was approved by the Sejong General Hospital Institutional Review Board (2017-1744).

## Results

### Overview of the CANE Platform

**Figure 5:**
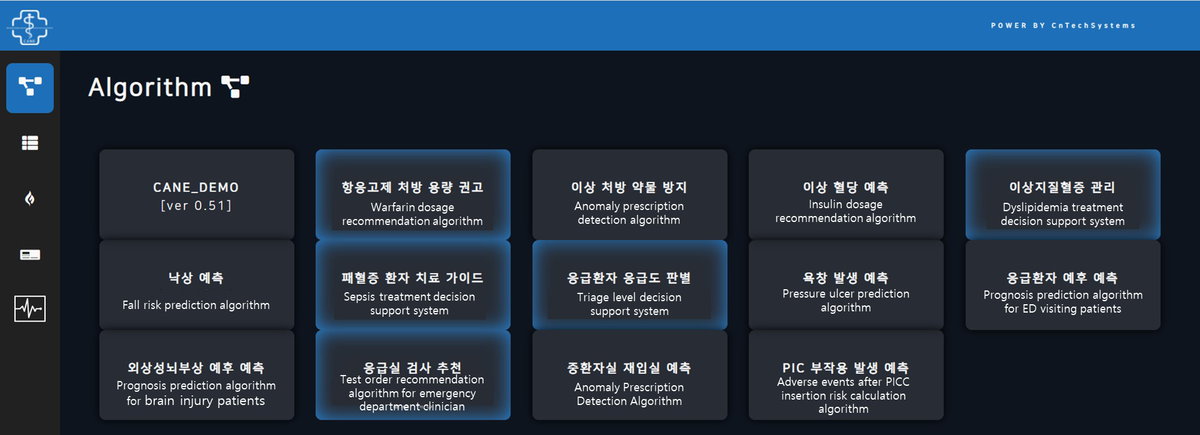
CANE dashboard of the demo web page. ED: emergency department; PICC: peripherally inserted central catheter.

Currently, eight algorithms are deployed to the CANE platform; however, the research consortium intends to deploy 11 algorithms by December 2022 (Table 1). When the CANE system is integrated with an EHR system, the algorithms mounted on the CANE system can be directly called through the RESTful application programming interface (API). The CANE system also provides a web interface (Figure 5). For the web session, this study focused on the CANE platform and representative use cases of both knowledge- and nonknowledge-based CDSSs, rather than discussing all of the deployed algorithms, which would extend beyond the objectives of this paper.

**Table 1:** Details of the developed algorithms for clinical decision support systems (CDSSs).

Algorithm name		Objectives	Target patients
**Nonknowledge-based CDSSs**			
	Anomaly prescription detection algorithm	To determine whether the prescription has potential information errors	Patients prescribed the following medications: heparin, Humulin, RI^a^, and potassium
	Test order recommendation algorithm for emergency department clinician	To recommend a prescription that is expected to need an examination based on the patient’s medical record, but omitted	Patients visiting the emergency department
	Triage-level decision support system	To determine the acuity of patients visiting the emergency department	Patients visiting the emergency department
	Emergency department visiting patients’ prognosis prediction algorithm	Algorithm for screening patients with a possibility of poor prognosis among patients visiting the emergency department	Patients visiting the emergency department
	Brain injury patients’ prognosis prediction algorithm	Algorithm for screening patients with a possibility of poor prognosis among patients visiting the emergency department	Patients with traumatic brain injury
	Fall risk prediction algorithm	To improve patient safety by predicting patients with a high risk of falls	Inpatients
	Pressure ulcer prediction algorithm	To calculate the risk of pressure ulcer, allowing for preventive action and early detection	Inpatients
**Knowledge-based CDSSs**			
	Warfarin dosage recommendation algorithm	To recommend an appropriate dose of anticoagulant in consideration of individual patient characteristics and drug response	Patients prescribed warfarin
	Insulin dosage recommendation algorithm	To recommend an appropriate dose of insulin in consideration of individual patient characteristics and response to previous insulin administration	Patients with diabetes mellitus
	Dyslipidemia treatment decision support system	To integrate and represent knowledge of dyslipidemia treatment guidelines	Outpatients who require treatment for dyslipidemia
	Sepsis treatment decision support system (SepsTreat)	To screen sepsis patients and provide a sepsis treatment guideline	Inpatients and patients visiting the emergency department who require treatment for sepsis

^a^RI: regular insulin.

### Use Case 1: Sepsis Treatment Decision Support System

Sepsis is a syndrome caused by infection, which is a significant public health problem that results in a patient’s death without appropriate and timely treatment [22]. As the importance of early recognition and appropriate treatment is well defined, many researchers have attempted to develop early detection methods that may predict the outcomes of patients with sepsis.

**Figure 6:**
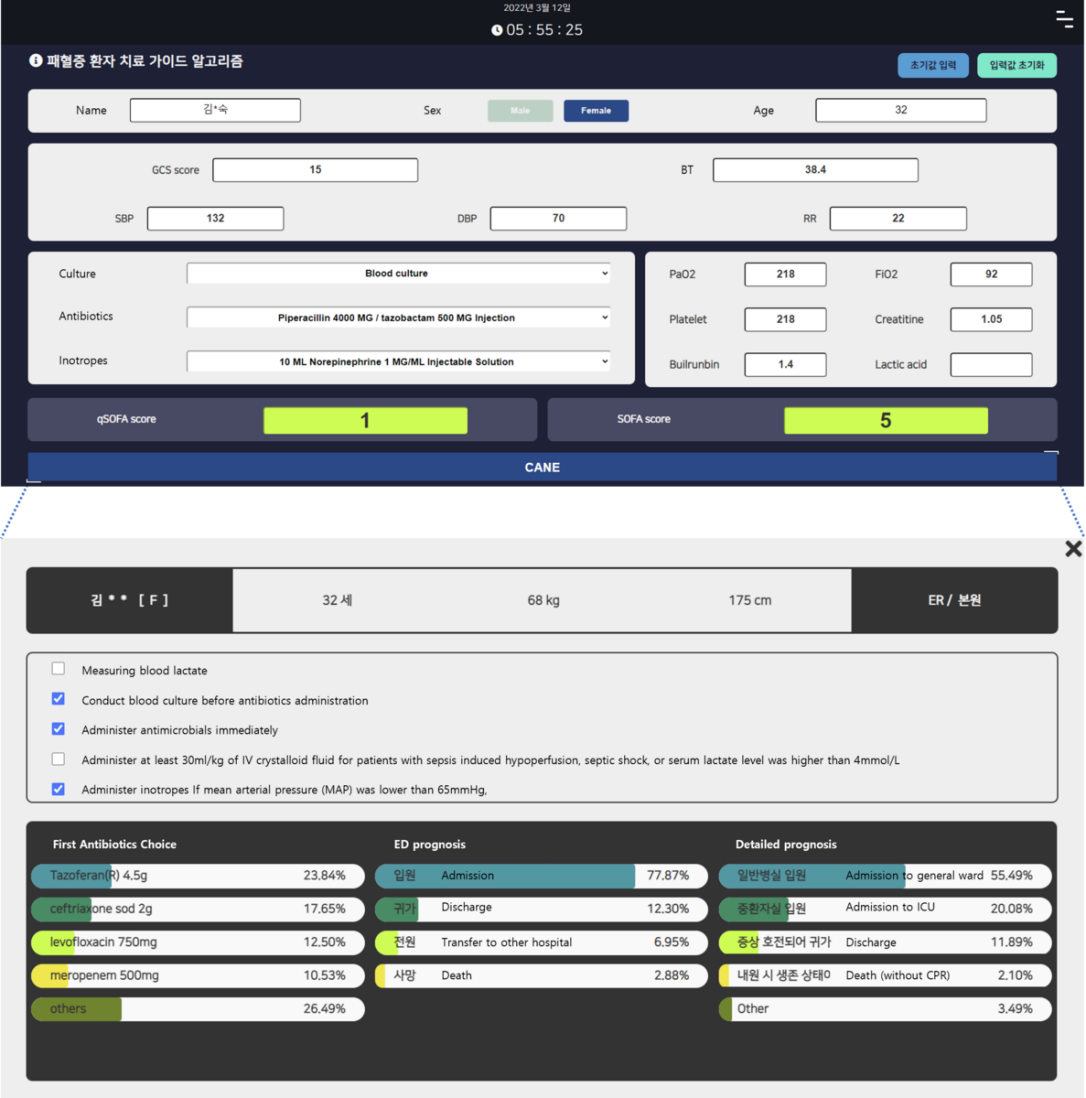
Screenshot of the sepsis treatment decision support system (SepsTreat) algorithm.

We developed a sepsis treatment decision support system (SepsTreat) as one of the knowledge-based CANE algorithms (Figure 6). This algorithm is rules-based, and it provides recommended treatment guides when detecting sepsis patients. Sepsis knowledge and treatment information are based on Sepsis-3 Guidelines [23]. Patients who show signs of organ dysfunction caused by infection are defined as sepsis patients. For SepsTreat, a sepsis patient is one whose body temperature is above 37.5°C and has been recommended a blood culture test or antibiotics. Organ dysfunction is checked using the Sepsis-related Organ Failure Assessment (SOFA) score, which cannot be calculated immediately because some score components require laboratory testing results. However, Quick SOFA (qSOFA) is a new method that helps physicians quickly assess patients suspected of infection, and offers investigative leads concerning suspected organ dysfunction. qSOFA was recently added to the Sepsis-3 Guidelines to supplement the complex SOFA score. The qSOFA score monitors only three components: systolic blood pressure, respiratory rate, and mentality.

A prototype of SepsTreat runs on the web as a separate instance paired with an EHR system. SepsTreat intakes component values to determine sepsis by calculating qSOFA and SOFA scores using the Glasgow Coma Scale score, systolic blood pressure, diastolic blood pressure, respiratory rate, body temperature, alveolar oxygen partial pressure, fraction of inspired oxygen, platelet count, creatinine, total bilirubin, lactic acid, blood culture order, antibiotics order, and vasopressors or inotropic medication orders. After patient examination, the physician enters each component value into SepsTreat. If the patient is in a septic condition, SepsTreat provides the recommended treatment. When the recommendation is displayed, practices that have already been completed are marked to emphasize treatments that have not yet been processed. Information retrieved from the OMOP-CDM database is also presented to help physicians manage patients. This information includes statistics of prescribed antibiotics for sepsis patients and the statistical results of sepsis patient outcomes. Physicians in a secondary hospital or clinic can easily access tertiary information when using the CANE system by presenting statistical results from their CDM database. In the prototype version, a single center’s practice statistics are presented. The final version will include patterns of prescribed antibiotics for sepsis patients from different hospitals and different outcomes. 

### Use Case 2: ED Patient Triage Algorithm

We incorporated a deep-learning prediction algorithm to extend CANE’s flexible boundaries. Kwon et al [24] created this algorithm, which calculates triage and acuity scores for ED patients. This algorithm also predicts hospital mortality, critical care, and hospitalization metrics using information from the triage stage (eg, age, sex, chief complaint, time from symptom onset to ED visit, arrival mode, trauma, initial vital signs, and mental status). Physicians can predict patients’ outcomes before the point of examination using this algorithm. From the results, physicians can deliver appropriate management before the patient’s deterioration, or they may opt to hospitalize patients sooner than otherwise expected to stabilize those with severe conditions.

To integrate this algorithm into the CANE platform, we coded the algorithm’s variables based on the concept ID of OMOP-CDM and retrained the algorithm using OMOP-CDM data. In the prototype of this algorithm, if a user enters input variables into the CANE platform, it presents each prediction result with a possibility and predefined risk score. Risk scores were determined in advance by researchers using statistical calculations. The resultant ED patient triage algorithm is a good example of how the CANE platform can integrate machine-learning algorithms with minimal integration effort. 

## Discussion

### Principal Results

In this paper, we introduced the CANE platform, which supports the deployment of various types of CDSS models. Our system was primarily developed to facilitate the deployment of medical artificial intelligence (AI) algorithms developed by the CANE Consortium. However, the platform can also be used as a pipeline that integrates CDSS and hospital developers who need to collaborate on tools. Furthermore, our platform supports the transformation of algorithms developed using R and Python into C#, which was required by the CANE platform. Considering that R and Python are widely used programming languages [25], our platform could contribute to overcoming the chasm between development and deployment of medical AI.

### On-Demand Intervention Type

We adopted “on-demand interventions,” which is a form of noninterruptive intervention, as the approach used by the CANE system to provide decision support. According to a recent meta-analysis, interruptive intervention is the dominant form of a CDSS that has been applied and utilized in clinical practice [26]. A CDSS applied in an interruptive manner not only distorts the clinical workflow but also reports unintended consequences such as alert fatigue, which is a known factor that hinders the CDSS from achieving its purpose [4]. Hence, interruptive intervention must be applied carefully in a limited purpose [27]. Therefore, we chose on-demand intervention as the basic intervention format. By subsequently applying the CDS Hooks to the CANE system, each CDSS can readily be invoked by various intervention methods according to the clinical workflow.

### Comparison With Prior Work

Despite evidence indicating that medical AI and CDSS can improve the efficacy and safety of health care delivery systems [28-30], the present situation still seems to be far from this goal. Studies have been conducted to address interoperability issues and overcome the chasm between CDSS model development and widespread deployment. Khalilia et al [18] provided convincing answers to account for this gap in terms of web services based on a service-oriented architecture. They presented a streamlined architecture that facilitates predictive modeling using OMOP-CDM structured data sets and deployed the model into a clinical workflow using HL7 FHIR. More recently, Gruendner et al [19] introduced a sophisticated and comprehensive platform that included model development, deployment, and security with a graphical UI based on OMOP-CDM and HL7 FHIR standards. Unberath et al [31] suggested an operational CDSS case to predict relapses in patients with melanoma using OMOP-CDM and the REST API.

The CANE system is distinguished from these previous works in that it provides data-driven decision support from other institutions. Thus, clinicians may refer to summary reports regarding similar situations using interconnecting integration modules at the point of care. Considering that clinicians prefer to make decisions based on peer opinions as well as CDSS information, this function is gaining importance from a behavioral science perspective [32]. Because various institutions participating in this study have constructed OMOP-CDM databases, all processes, including data queries, data analyses, and reports generation, are conducted within the hospitals’ firewalls. Only summary reports are transmitted to the requesting institutions. Raw patient data are avoided via an interconnecting integration module to minimize security risks.

### Model Deployment Determining Pipeline

The CANE Consortium should establish a pipeline that determines the installation of a nonknowledge-based CDSS in the CANE system, with the aim of distributing a model to other institutions. Several studies have reported that performance indicators evaluated using external data are statistically significantly lower than those evaluated using data from institutions where an AI model was developed. This could be attributed to the “Cloud of Context” issue [33]. Variations in clinical workflow, available resources, and patient characteristics among institutions hinder the generalizability of an algorithm. Hence, external validation using data from a target institution before applying AI models is an effective way to not only adjust the expectations on the model but also to prevent potential patient safety issues due to the algorithm. Therefore, we suggest that information regarding external validation must be included in the evaluation pipeline to determine whether a specific algorithm should be installed into the CANE platform.

### Limitations

This study has the following limitations. The CANE platform does not embrace the machine-learning algorithms developed from the ATLAS platform, which is a widely used web-based service for building machine-learning models within the OMOP-CDM ecosystem. Second, the Consortium did not investigate the performance indices of each algorithm using either internal or external data, or the usability of the CANE platform. Further evaluation is needed in a subsequent study. Third, it is common that the performance of machine-learning algorithms differs when they are applied to other organizations. Finally, CQL and CDS Hooks, which are standards recently highlighted in the field of medical informatics, were not reflected in our system.

### Conclusions

We introduced the CANE platform, which adheres to medical informatics standards (OMOP-CDM and HL7 FHIR). This system provides summary data on the treatment patterns of other institutions that could aid physicians’ decision-making. Moreover, concerns regarding potential privacy issues are minimized by transmitting summary data rather than individuals’ raw heath data. 

## Abbreviations

AI: artificial intelligence
API: application programming interface
CANE: common data model–based intelligent algorithm network environment
CDM: common data model
CDSS: clinical decision support system
CQL: Clinical Quality Language
ED: emergency department
EHR: electronic health record
FHIR: Fast Healthcare Interoperability Resource
HL7: Health-Level 7
JSON: JavaScript Object Notation
OMOP: Observational Medical Outcome Partnership
PCORnet: Patient-Centered Clinical Outcomes Research Network
qSOFA: Quick Sepsis-related Organ Failure Assessment
SepsTreat: sepsis treatment decision support system
SNOMED: Systematized Nomenclature of Medicine–Clinical Terms
SOFA: Sepsis-related Organ Failure Assessment
UI: user interface
